# Quinine in Malaria, Excluding the Simple Intermittents*Read before the Clinical Society of St. James Dispensary, January 1, 1898.

**Published:** 1898-02

**Authors:** J. G. Van Marter

**Affiliations:** Savannah, Ga.


ATLANTA

Medical and Surgical Journal.

Vol. XIV. FEBRUARY, 1898. No. 12.

L. B. GRANDY, M.D., DUNBAR ROY, M.D., EDITORS.

M. B. HUTCHINS, M.D., BUSINESS MANAGER.

### *ORIGINAL COMMUNICATIONS.*

### QUININE IN MALARIA, EXCLUDING THE SIMPLE INTERMITTENTS.*

By J. G. Van MARTER, Jr., M.D.,

Savannah, Ga.

There is a vast difference between the action of quinine in the intermittent malarial fevers and its action in the continued malarial fevers, "the malaria subcontinua typhoidea" of the Roman school, the malarial cachexia and the debatable "terra incognita" of malarial toxemias seen in hot paludal countries.

This paper should properly have been preceded by papers and discussions on the etiology, pathology and clinical varieties of the malarial toxemias as seen by us in this climate. This would have enabled me to classify the action of quinine according to the pathology and clinical picture of each case. Such a discussion as this is an eminently fitting and proper one in our locality, and it is high time that a protest be issued against the general acceptance of the views of Osler, Thayer, and other Northern clinicians on the specificity of quinine.

* Read before the Clinical Society of St. James Dispensary, January 1, 1898.

Before entering into an argument upon the specific action of quinine I wish to state that while Thayer in his recent work on malaria distinguishes three types of the malarial parasites, the tertian, the quartan and the parasite of estivo-autumnal fever, I believe that it is safe to assert that there is more than one variety of parasite in estivo-autumnal malaria, and that these parasites have entirely different action as regards the manufacture of toxins.

Under quinine the forms of the ordinary cycle of development disappear rapidly from the peripheral circulation, but the crescentic and ovoid bodies remain a much longer time, sometimes even for months.

In this paper I shall abstain entirely from a discussion of the treatment of malaria either preventive or curative, but shall confine myself as closely as possible to the action of quinine, and we shall see whether quinine is a true specific or preventive of the various forms of malaria with which I have been acquainted, both here and in Rome.

Osler, Thayer, Councilman, and others in the North speak truthfully when they say that quinine is a true specific against the malarial parasite and the malarias which have come under their observation, but as these gentlemen have not studied malaria in its true home, in a climate fitted for the development of the most virulent parasites, with infections occurring the year around, they are not fitted by either experience or observation, to settle the question as regards the action of quinine in the malaria that we see here; this applies to the entire region south of Charleston, and the Gulf coast.

In the first place let us see whether quinine is the best preventive of infection. It is so stated by Northern writers, English, French and German authorities, but is this the case with those who live in the regions infested by the severer varieties of malaria? No; they find by experience that quinine *cannot be taken indefinitely the year round* in doses sufficient to kill the parasite as fast as it develops, and various peoples have adopted different measures to prevent infection, which prove *better for long-contined use.* In some parts of Italy they find that a strong decoction of fresh lemons will prevent infection, while in other regions of Italy the continual use of small doses of arsenious acid acts well; while in India, Assam, and Cochin-China, the natives working in the rice fields and subject constantly to severe infection find that opium will prevent malaria where quinine fails. This to me is very suggestive, being far better evidence upon which to base conclusions than the mere hypothesis of Northern observers, or the passing observations of travelers; of course, they can take quinine for a few weeks or months.

Now coming to the treatment of malaria, and of course I am always speaking of the severe infections and leaving out of the question the intermittent fevers, is quinine a true specific? No, there are cases innumerable in which the patient would die did we not add other potent drugs to our quinine, or for a time at least attach more reliance on other drugs than quinine, that we are inevitably lead to the conclusion that although a true specific against the plasmodium of tertian and quartan fever, it is not a specific or antidote to the parasite of the more severe continued malarias and the toxins generated by them. With patients in whom the microscope has shown the disease to be malaria in any of its forms, quinine is a specific in all those with intermissions or with marked remission; not so however where the fever is continued or in those malarias with but little temperature.

In the continued fevers towards the last stages, marked remissions are apt to occur (in the milder cases); here again quinine becomes a specific. There are however exceptions even to this rule, for it is no uncommon thing to see a patient with intermittent fever to whom quinine has been properly administered have a distinct malarial paroxysm with the ears ringing from quinine.

Dr. Plehn, a German physician practicing in the Cameroons, on the west coast of Africa, and in what is probably the most malarious region on this earth, has observed that quinine is a good preventive, and the best for treatment in newcomers and those not long resident in that region, but in spite of five grains per diem, practically *all* foreigners get the fever, and the large majority die of it sooner or later. In all these cases, with the exception which I shall hereafter note (the hematurias), quinine is given in enormous doses, with calomel (which, by the way, is never omitted) and stimulants, but while the actual paroxysm is overcome by the quinine (if the case be seen in time, or not too malignant), the spleen remains large, *the crescents remain in the blood*, and malarial anemia sets in. What does this interesting observation show?

1. That quinine is a specific against the protozoon of tertian or quartan malaria.

2. That it inhibits for a time the development of the protozoon of pernicious malaria, but does not kill it, nor in time, even with quinine constantly taken, prevent its development, *every time the patient catches cold*, or is exposed to a particularly severe contagion.

3. That quinine alone has no action on the toxin produced by grave malarias, over which calomel has twice the potency (at least in full physiological doses).

4. That quinine, even as a prophylactic, cannot be indefinitely taken.

5. That quinine has no effect whatsoever on malarial anemia (really a chronic toxemia).

Another very interesting form of protozoal malaria, beriberi, is not cured by quinine, although slightly benefited for a time, *if the febrile manifestations are sharp.* In this malignant form of disease, the pigment bodies seen in our own malarias, are deposited in the brain and other nerve tissues, and these pigmented bodies, either before or after degeneration, produce a toxin absolutely unaffected by quinine. In the recent epidemic at the Insane Hospital, Tuscaloosa, Ala., reported by Dr. Bondurant, in the *New York Medical Journal*, it is stated that quinine failed in every case to do any good. It is to be regretted that special work on the etiology was not done, and establish the fact of direct relationship of the sporozoons supposed to cause beriberi.

Still another variety (more correctly complication) of malaria, hemoglobinuria, is made worse by quinine. I believe that the great majority of those practicing in countries where severe malarias exist will confirm the observation that quinine makes it worse. Thayer admits that quinine never shortens an attack of hemoglobinuria, but says it prevents a recurrence, this latter being an assertion without any warrant of experience, and I know it to be wrong. Quinine is a *frequent* cause of hemoglobinuria, and after one attack, if quinine be taken, is very apt to *cause the very condition which Thayer says it will prevent.*

In my experience the cases of malaria (as proven by the microscope) in which quinine failed to cure, hence did not act as a specific, have been confined to two types. First, and most common, severe malaria subcontinua typhoidea (an estivo-autumnal form) where the fever ran along for days with very slight remissions, and, second, those irregular forms, sometimes seen, where, with undoubted malaria, the fever of a continued type is low, seldom above 102, the symptoms presented are those of a profound toxemia resembling uremia; suppression of urine, jaundice, delirium, subsultus, without chills or paroxysms of any kind.

I will observe here that there are cases of both types, above mentioned, that would surely die, did we not add other drugs to our quinine, and that a large proportion of cases for a time at least are better off without it. We must recognize that in these types we have a toxemia to deal with, and it is just this toxemia which I claim is unaffected by quinine.

In treating these very severe malarial toxemias as we see them in the country or plantations just out of town, or if nearer, only in the suburbs, or on river sailors, we are placed at a great disadvantage as regards doing the best possible for our patients. Perhaps in these very cases if our patients could be moved away from an atmosphere whence constant reinfection is taking place, and taken to a good hospital like the Johns Hopkins in Baltimore, where the poor even can obtain luxuries, and under skilled, trained nursing, perhaps in *such* cases quinine might help our patient *if* proper eliminative treatment were added (for it would not without it); but how is it with us—the patient in a miserable hut or poor farmhouse, not a bath-tub in miles, no clean bed linen, no decent drinking-water, no chance of proper food or good nursing, and an unalterable opposition to hospitals in general? It is in these cases, gentlemen, that we have to practice, for it is only amongst such that we see most of our severe malarias, and if quinine were a specific, they would all be cured before we ever see them. They all take plenty of quinine dissolved in water, and with it, calomel.

I have tried, in the severer cases, quinine intravenously, and must say, that it does act well, that it is the only way to give it in the severest forms, but it does not shorten the course of the fever; it seldom breaks it up, as it should if it were a specific.

I am one of those who believe that quinine is seldom properly administered. It is not the amount, but the way you give it that counts; give it with an acid, if the stomach will stand it, or else, if you still desire to give it by the mouth, give it in the effervescing form recommended by Burney Yeo. I quote from him as follows: "We may state in this connection, that we have found the efficacy of quinine in febrile states very much influenced by its mode of administration. If we prescribe the quinine dissolved in citric acid, and given in effervescence by adding it to an alkaline mixture, doses of two or three grains exert powerful antipyretic influence, far greater than that obtained by the same quantity of quinine given in the dry state." "We have seen abundant reason to believe that, in infective fevers, if quinine be given in saline solutions, it is the most active and reliable antitoxin we at present possess." *

The use of a strong decoction of lemon, in the early morning is a very useful remedy, but I cannot branch out to speak of its action, because that would be exceeding the limits of my subject.

There is one way of giving quinine by the mouth, of particular efficacy in many of the severe varieties, and that is Warburg's tincture, and to my mind it is a most excellent medicine.

One very strange observation that I have made is worth relating. I had a patient with occasional severe attacks of malaria, who for some reason or other never seemed to get the physiological effects of quinine, in other words he never had ringing in the ears; thinking that the quinine was not being absorbed properly, although I had given it in various ways, I gave him several hypodermics, by the method I shall further on describe, and failing in this I put him on big doses of the Warburg's tincture, and strange to say, *one ounce of Warburg's Tincture made his ears ring.* This extraordinary phenomenon has often been a source of perplexity to me, and in reasoning about it, have come to the conclusion that something in this compound may act in a slight measure as an antitoxin, or in some way so modify the chemistry of the blood, as well as the

*Burney Yeo, Clinical Therapeutics, Vol. II., page 637.

activity of the glandular and eliminative system, *as to give quinine a chance.* Is it not true, perhaps, that quinine meets with resistance in the blood which is in some way modified by that complicated mixture—Warburg's tincture? Gentlemen, this is a very interesting subject upon which much remains to be known.

Warburg's tincture should always be given, as recommended by the experienced practitioners in India, after a brisk purge, undiluted, in doses of half-ounce, all drinks withheld, repeated in three hours, and the patient carefully rolled up in blankets to encourage the profuse aromatic perspiration which follows. It is one of the most powerful diaphoretics known; it is also a diuretic, a stimulant and a purgative. I always follow its use by opium and small doses of whisky; at least never omit the opium, which I believe acts most happily. Recently I have used the powdered Warburg's tincture put up in elastic capsules by Messrs. Parke, Davis & Co., an elegant preparation, far more agreeable to the patient than the liquid, but I am far from convinced that it compares to the liquid preparations obtainable in England, for our American preparations of the liquid do not act as well as the English.

Quinine by the rectum I do not favor, and shall not speak of to-night, nor shall I say anything about inunctions, for that is an uncertain way of giving it, but of the hypodermic method I am a great advocate. In giving quinine subcutaneously let me urge you to use it in free solution, and not to stick to your small hypodermic syringes. I am now using what is usually called an "antitoxin" syringe with a 16 c. c. capacity. By making a very dilute solution, more quinine is promptly absorbed and there is absolutely no danger of abscess or painful inflammations. You should not use an acid to dissolve the quinine as is advised by most writers, for it is not necessary and is very painful. The dihydrochlorate and hydrobromate of quinine are the two salts best adapted for such use, and also for intravenous injection; the water should be hot, about 100° F., and the needle sharp. Whenever in any case of malaria the gastric symptoms are marked (and this is frequent), use the hypodermic method in the commencement; you are then *sure* that the patient is getting all the quinine you want him to have promptly and without additional burdens on the stomach.

I have never seen but two abscesses (and they were not in my practice) from hypodermic injections of quinine, one due to an excess of acid, the other to a filthy syringe. Don't inject in the arms. The belly wall is a very handy place to inject your solutions, and never bothers the patients like it does in the thighs or back. Use from eight to ten grains at each injection, and if the quinine does not work promptly don't pin too much faith in it, nor that absurdity called the therapeutic test—a relic of barbarity.

Intravenous injections of quinine you are all more or less familiar with, at least as regards technique, which is simple; but I find a good deal of hesitation amongst a great many physicians as to its use, fear of its difficulty, of slipping up in asepsis, or admitting air in the vein, all points easily avoided and overcome. Having been brought up, you might say, on this method of using quinine, and having seen its development in the Santo Spirito Hospital Rome, in the service of Baccelli, I have had very good opportunities of seeing it practiced. Here as elsewhere in this paper, I shall have to remind you that as this is not a paper on the treatment of malaria, I must refrain from describing the method, its indications, advantages, but simply its action.

We get by an ordinary injection what, for the blood, is a very large amount (fifteen grains), and I honestly believe that if we could see our cases early enough, all cases of pernicious malaria of a fulminating type could be saved; but alas, we seldom see them early, for such cases come in town from the country *in extremis;* this, at least, is the common experience in Rome. Once the plasmodia have had time to fully manufacture their toxin, it is too late to rely on quinine. As however I have seen several cases recover after intravenous injections in the last stages, you may well ask how it is that they did not die too. Gentlemen, I attribute a good share of *some* (not all) of the recoveries to the happy effects of quinine, but some are due to the salt solution injected at the same time. It has never yet been done by control experiments, but I have no doubt that if you gave some of these cases a large intravenous injection of normal salt solution, say twenty ounces, and no quinine, you would get *as good results as you could by quinine.* I know from experience that this will start secretion in the kidneys—the only channel by which the poison escapes in this condition. Any observing man whose misfortune it has been to have a number of severe pernicious malaria cases in his practice, will agree with me, when I state that *if you can* set up diuresis, sweating, and purging, while vigorously stimulating the patient, he is apt to live, quinine or no quinine, and on the other hand, without this, but all the quinine you please, the patient will die—it is the common experience we have with every poison from malaria to rattlesnake bite.

Let us not say a thing is so because the books written by great teachers say it is so. Let us observe, reason, and then if we are not satisfied of the accuracy of a statement, let us say so. The last words have not yet been spoken on the specific action of quinine in malaria (in our climate), and in the same breath I will say *the* book on malaria has not yet been written. I know quite well that there will be wailing and gnashing of teeth over the presumptuousness which could question the specificity of quinine, but the truth will out, and I feel confident that many will agree with my views on this subject.

To summarize briefly my conclusions are:

1. As a preventive quinine will not do for those who are compelled to live indefinitely in a severe malarial climate; in time, acting as a vaso-motor poison.

2. Quinine acts nearly as a specific in all malarial fevers characterized by intermissions or well-marked remissions, but fails in the continued fevers, those with typhoid-like symptoms, those malarias without temperature, and the cachexias and anemias due to malaria.

3. Proving thus that quinine is a poison to the plasmodium itself, but useless against the toxin manufactured by it.

4. That Warburg's tincture in the last condition has an action not yet understood on the toxin (or the eliminative system) by which the system is put in condition to benefit by the quinine.

5. That quinine should never be used in hemoglobinuaria, or given subsequently, to one who has suffered from it, being liable to bring about a recurrence of the condition.

6. Only those living in regions of severe malarias can become competent to settle these questions *pro* or *con.*

